# ESR essentials: MRI of the knee—practice recommendations by ESSR

**DOI:** 10.1007/s00330-024-10706-7

**Published:** 2024-03-27

**Authors:** Anagha P. Parkar, Miraude E. A. P. M. Adriaensen

**Affiliations:** 1 Present Address: Radiology Department, Haraldsplass Deaconess Hospital, Postboks 6165 Posterminalen, 5892 Bergen, Norway; 2 Department of Clinical Medicine, Faculty of Medicine and Dentistry, University of Bergen, Postboks 7804, 5021 Bergen, Norway; 3 Department of Radiology, Zuyderland Medical Center, Henri Dunantstraat 5, 6419 PC Heerlen, the Netherlands

**Keywords:** Knee, Magnetic resonance imaging, Cruciate ligaments, Menisci, Evidence-based practice

## Abstract

**Abstract:**

Many studies and systematic reviews have been published about MRI of the knee and its structures, discussing detailed anatomy, imaging findings, and correlations between imaging and clinical findings. This paper includes evidence-based recommendations for a general radiologist regarding choice of imaging sequences and reporting basic MRI examinations of the knee. We recommend using clinicians’ terminology when it is applicable to the imaging findings, for example, when reporting meniscal, ligament and tendon, or cartilage pathology. The intent is to standardise reporting language and to make reports less equivocal. The aim of the paper is to improve the usefulness of the MRI report by understanding the strengths and limitations of the MRI exam with regard to clinical correlation. We hope the implementation of these recommendations into radiological practice will increase diagnostic accuracy and consistency by avoiding pitfalls and reducing overcalling of pathology on MRI of the knee.

**Clinical relevance statement:**

The recommendations presented here are meant to aid general radiologists in planning and assessing studies to evaluate acute and chronic knee findings by advocating the use of unequivocal terminology and discussing the strengths and limitations of MRI examination of the knee.

**Key Points:**

*• On MRI, the knee should be examined and assessed in three orthogonal imaging planes.*

*• The basic general protocol must yield T2-weighted fluid-sensitive and T1-weighted images.*

*• The radiological assessment should include evaluation of ligamentous structures, cartilage, bony structures and bone marrow, soft tissues, bursae, alignment, and incidental findings.*

## Key recommendations

• The knee should be examined with images in sagittal, coronal, and transverse planes so that crucial pathological findings are not missed or misinterpreted (Level of evidence: moderate).
• The general protocol must yield both T2- and T1-weighted images; the latter used to differentiate between causes of bone marrow pathology and to detect microfracture lines, while (fluid-sensitive) T2-weighted images are used for oedema in acute trauma and chronic knee pain (Level of evidence: low).
• The radiologist’s assessment should include evaluation of all ligamentous structures, menisci, cartilage, bony structures, bursae, joint alignment, soft tissues, and incidental findings, regardless of a narrow clinical indication (Level of evidence: moderate).

## Introduction

MRI of the knee is a common examination performed both in outpatient clinics and for hospitalised patients [1]. The indications vary extensively. In the knee, MRI is especially useful in assessment of soft tissues after acute (sports-related) injuries. In cases of chronic knee issues, the range widens to encompass chronic overuse and inflammation. Neoplasms and osteomyelitis are also indications in knee imaging, although not specific to the knee itself [2]. Often the indication given is non-specific and for initial assessment a radiology department needs a primary standard MRI protocol for the knee. A well-adjusted protocol can detect most knee pathologies, and the few that require further examinations can be referred or recalled for additional sequences [3]. The European Society of Musculoskeletal Radiology (ESSR) sport imaging protocol might be used as baseline [4].

The recommendations presented here are meant to aid general radiologists to plan studies for and to assess acute and chronic findings in the knee. The assessments of bone tumours, generalised bone marrow abnormalities, and/or osteomyelitis are not knee-specific entities and are thus not included in this paper. The following recommendations are considered good practice recommendations. They are meant as a “base camp” knee MRI. These recommendations should not prevent implementation of technical improvements or a more refined radiological evaluation. Although we do not recommend a structured report for knee MRI, the reporting of certain pathologies requires standardised assessments [5–7]. The aim herein is to improve the usefulness of the MRI report by understanding the strengths and limitations of the knee MRI exam.

## Recommendations

### MRI protocol

The examinations may be performed on both 1.5-T and 3-T scanners [8]. Recently, the technological development has made 3D imaging of the knee clinically feasible, and will lead to a decrease in artefacts, better imaging of cartilage, nerves, and bone shape, as well as faster imaging time, as one acquisition can yield image reconstructions in any plane [9]. Unless performing 3D imaging, the knee should be examined in three orthogonal planes: sagittal, coronal, and transverse. The sagittal plane is either oriented sagittal perpendicular to femoral condyles or slightly oblique in order to visualise the anterior cruciate ligament in its entirety on a single slice (as much as possible) [2]. Currently, the evidence-based consensus recommendations state that 4-mm slice thickness is acceptable in knee imaging, but slice thickness of ≤ 3 mm is preferable and available on most modern scanners [10]. It should be noted that 3D imaging in the knee can produce submillimetre image slices. In general, the following basic variations in sequences for knee MRI exist: T1-weighted images with or without fat saturation, T2-weighted images with varying fluid sensitivity, and proton density (PD) weighted [11]. A T2-Dixon sequence with post-processing may be used to replace fluid-sensitive PD, and T1 images and is efficient in bone marrow assessment [12, 13]. The use of deep learning techniques to improve signal to noise ratio and image quality in MRI will also improve diagnostics in the future, and shorten imaging time while maintaining image quality [9]. Commonly, T1 without fat saturation or PD-weighted images is used to evaluate anatomy, PD-weighted images for soft tissue, and fluid-sensitive T2 for bone marrow oedema detection, and T1 with fat saturation is only used in conjunction with contrast imaging [10]. Although the original T1 and later developed PD visually may appear the same, no studies comparing their efficacy in detecting bone marrow pathology and/or microfractures have been published; thus, PD images cannot replace T1 images. T1 images (can be replaced by Dixon T1-weighted images) are still considered obligatory in the standard knee MRI protocol [10, 14]. *Level of evidence of recommendation: 4/Low.*

Fluid-sensitive sequences can vary considerably and the choice of weighting must be adjusted according to scanner and local preference of the reporting radiologist [2]. In acute knee trauma, an MRI should always be accompanied by a radiograph (or computer tomography) of the knee to avoid missing small bony avulsions. If bone marrow pathology is detected first on MRI, radiographs or CT may be performed to supplement the MRI if the diagnosis is uncertain [15]. This should not be misinterpreted as “radiographs are unnecessary”, or that MRI of the knee should be the first modality regardless of clinical indication. *Level of evidence of recommendation: 4/Low.*

### Clinical indications for knee MRI

The optimal use of MRI is best decided in conjunction with key local stakeholders, but there are some overarching principles. MRI of the knee is indicated in acute knee trauma with suspected internal derangement and in the course of posttraumatic assessment [16]. It is also indicated in clinically unexplained chronic knee pain, when prior radiographs are non-diagnostic [17]. Some imaging centres vary their knee MRI protocols according to the clinical query. However, even though clinical history improves MRI diagnostics, important incidental or concomitant findings may be missed if all structures are not routinely evaluated [18].

### Basic image assessment

The assessment should include evaluation of the menisci (including roots), ligamentous structures and tendons, cartilage, bone and bone marrow, soft tissues, bursae, and femorotibial and patellar alignment, as well as any incidental findings. In the following section, when referring to T1, T2, or PD images, this also includes Dixon images which are derived from post-processing and reconstructed with various weightings [9].

#### Menisci

**Fig. 1:**
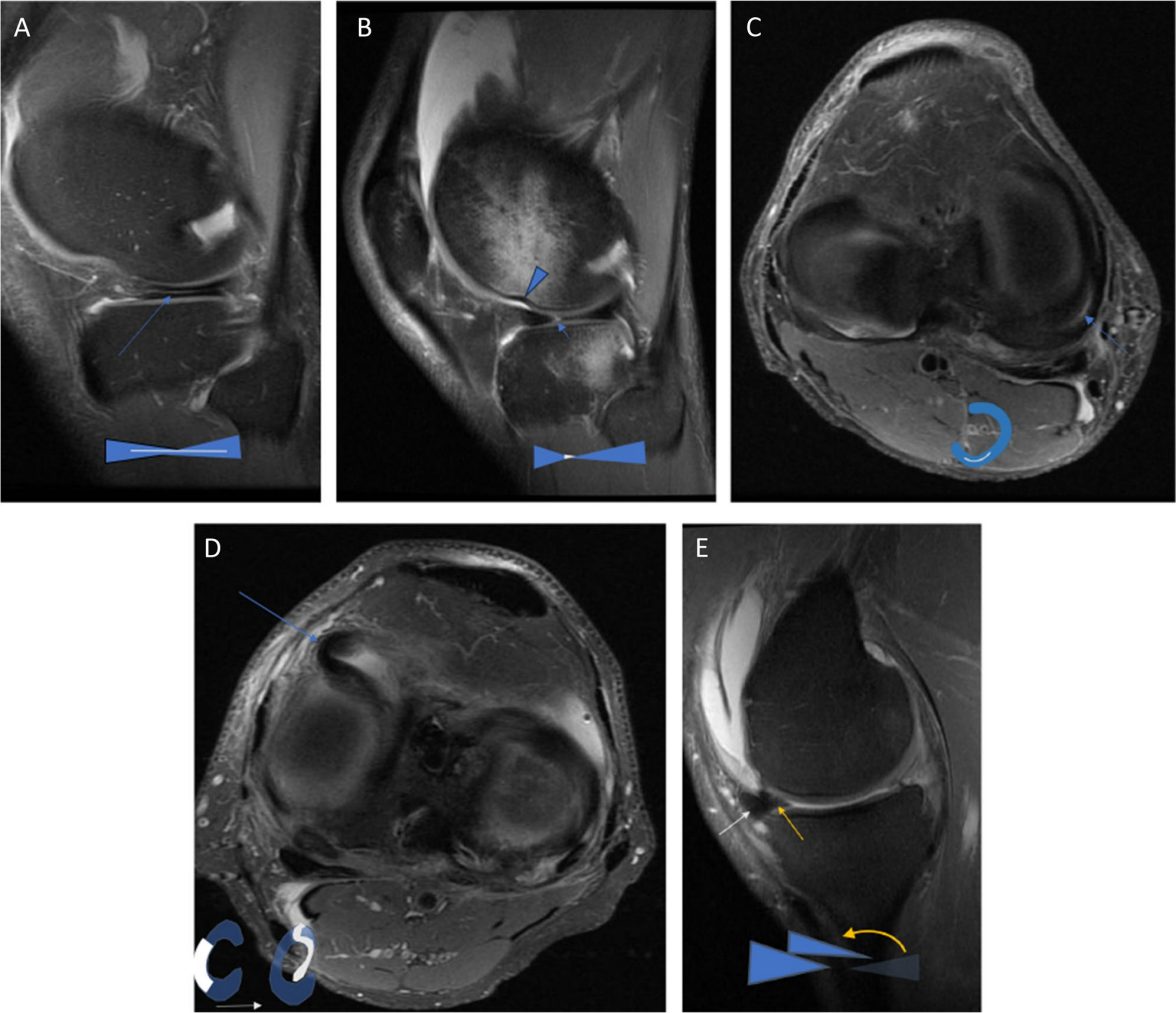
**A** PD (fat suppressed) sagittal image shows a horizontal high-signal intensity line in keeping with a chronic meniscal lesion (arrow). *Schematic drawings of the meniscal pathology in blue below.* **B** PD sagittal image shows a vertical (radial) high-signal line in keeping with an acute tear (arrow) which occurred after valgus trauma. The arrowhead points to a subchondral impression of the lateral femoral condyle typically occurring with ACL injury. In addition, diffuse high-signal areas are seen in femur and posterior tibia indicating oedema due traumatic bone bruise. *Schematic drawings of the meniscal pathology in blue below*. **C** PD transverse image shows a high-signal line depicting a vertical (longitudinal) meniscal tear in the medial meniscus (arrows). *Schematic drawings of the meniscal pathology in blue below.* **D**. PD transverse image shows a complex rupture, where part of the posterior meniscus is flipped anteriorly (like an s-shape, blue arrow), an unstable lesion which causes a locked knee. *Schematic drawings of the meniscal pathology in blue below.* **E** PD sagittal image shows two triangles on top of each other (yellow and white arrows) in the anterior region of the meniscus, but nothing in the posterior region, the so-called double anterior horn sign. *Schematic drawings of the meniscal pathology in blue below*

The lateral and medial menisci are semicircular fibrocartilage structures between the tibia and femur which function as shock absorbers and provide stability to the knee joint. Anteriorly and posteriorly, they both are attached to the tibia with meniscal roots. On MRI, menisci are best appreciated on coronal and sagittal images. They appear as homogenous low-signal triangular structures when normal on T1-, T2-, and PD-weighted images [19]. They can also be appreciated well on transverse images, where the semicircular form is seen. Pathology is seen as high signal lines that run horizontally, vertically (subdivided into radial or longitudinally), or complex (Fig. 1A–E). It is important to recognise lesions that require surgical treatment, as locking of the knee due to meniscal flaps or fragments damages the cartilage [5].

Meniscal tears and ruptures were previously described in the “red” and “white” zones, which were terminologies according to presumed vascularity of the meniscus. However, as the vascularisation changes (declines) over time, this terminology should be discarded [5]. Instead meniscal pathology location should preferably be described as anterior, mid-body, or posterior, combined with circumferential inner, mid, or outer parts [5]. The European meniscus consensus recommends only using the terms meniscal tears or ruptures after a “sufficient” prior knee trauma in the clinical history, otherwise the pathology should be reported as a mere meniscal “lesion” [5]. Further, a large tear (gap between flaps > 5 mm), a tear which may result in a locked knee, or a radial tear may be reported as an unstable meniscal tear, but they recommend refraining from defining stability of small meniscal tears on MRIs, as this is an arthroscopic diagnosis [5]. *Level of evidence of recommendation: 4/Low*.

Medial meniscal cysts are associated with meniscal lesions, but anterior lateral meniscal cysts are not [11]. Discoid meniscus is a normal variant where the shape is more circular than semicircular, and is prone to degenerative changes [20].

**Fig. 2:**
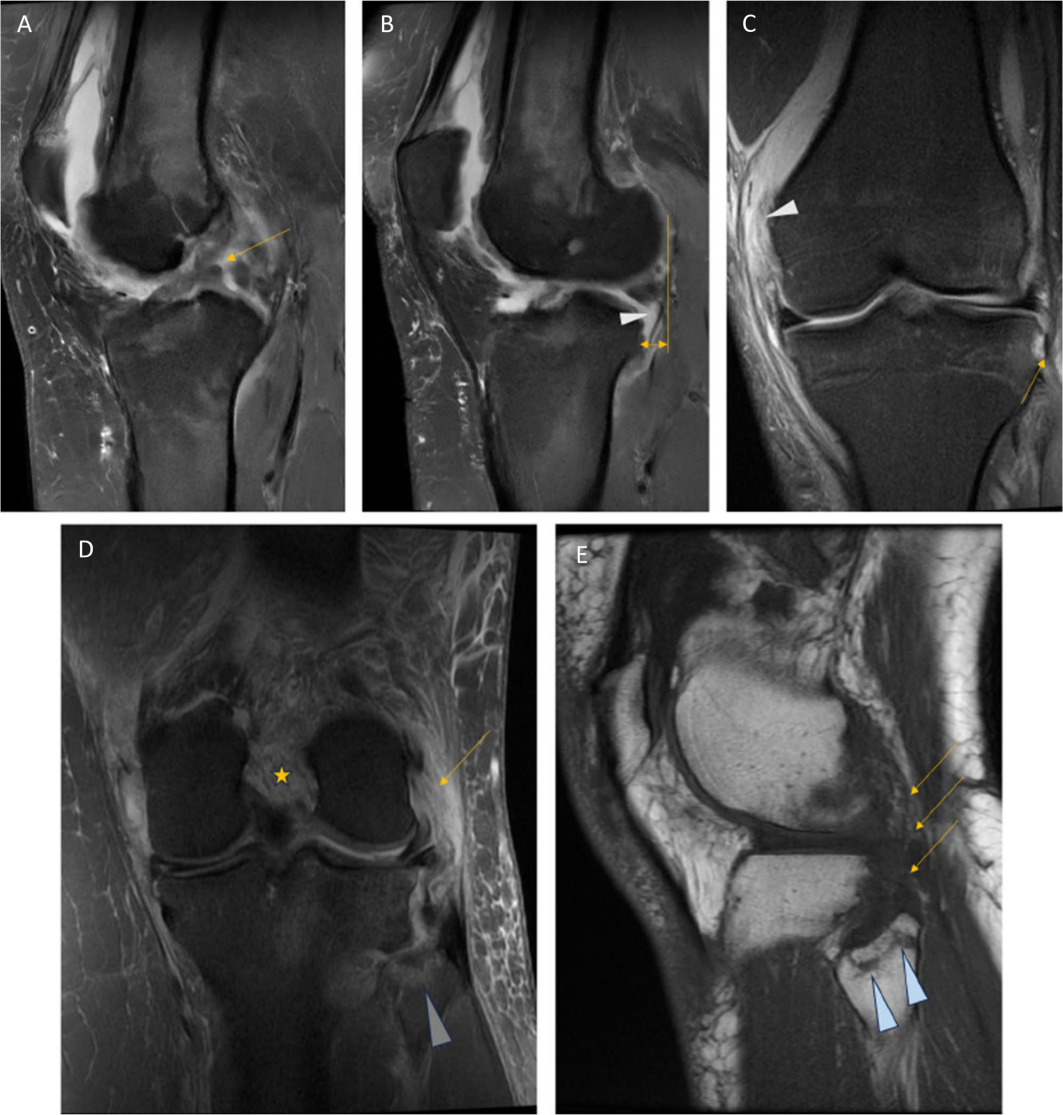
**A** PD sagittal image shows high signal in the ACL, and fibre discontinuity (arrow), which was proven to be a complete ACL tear. **B** Same knee as in A, indirect sign of anterior tibial translation in the lateral compartment, indicating ACL deficiency (yellow line). This patient also had a lateral meniscal posterior root rupture (arrowhead), where the attachment between the meniscus and the tibia is missing, i.e. a so-called ghost sign or empty meniscus sign. **C** PD coronal image, different case from the previous images (**A**–**C**), shows avulsion of the cortical rim, a Segond fracture (arrow), seen adjacent to a very small area of high-signal intensity in subchondral bone. Segond avulsion has a 95% association with ACL rupture (not shown); in addition, this patient also suffered a full-thickness MCL rupture at the femoral attachment (arrow head). **D** PD coronal image (different patient from **A**–**C**), asterisk shows ACL rupture, LCL rupture (arrow), high-signal intensity in fibular head (arrowhead). **E** Same patient as in **D**, T1 sagittal image shows pathology in the posterolateral corner (arrows) and avulsion fracture of the fibular head (arrowheads), much better appreciated on T1

Meniscal posterior root tears are an entity which has gained attention in recent years. Medial root tears are generally degenerative, and should preferably receive conservative treatment [5]. Compared to medial root tears, lateral root tears are usually traumatic, often occurring, in conjunction with a full-thickness ACL rupture, they are commonly missed on MRI. A missed meniscal root tear with subsequent non-treatment is associated with poorer clinical outcome in patients (Fig. 2C) [5, 21]. *Level of evidence 4/Moderate.*

#### Ligaments and capsular structures

**Fig. 3:**
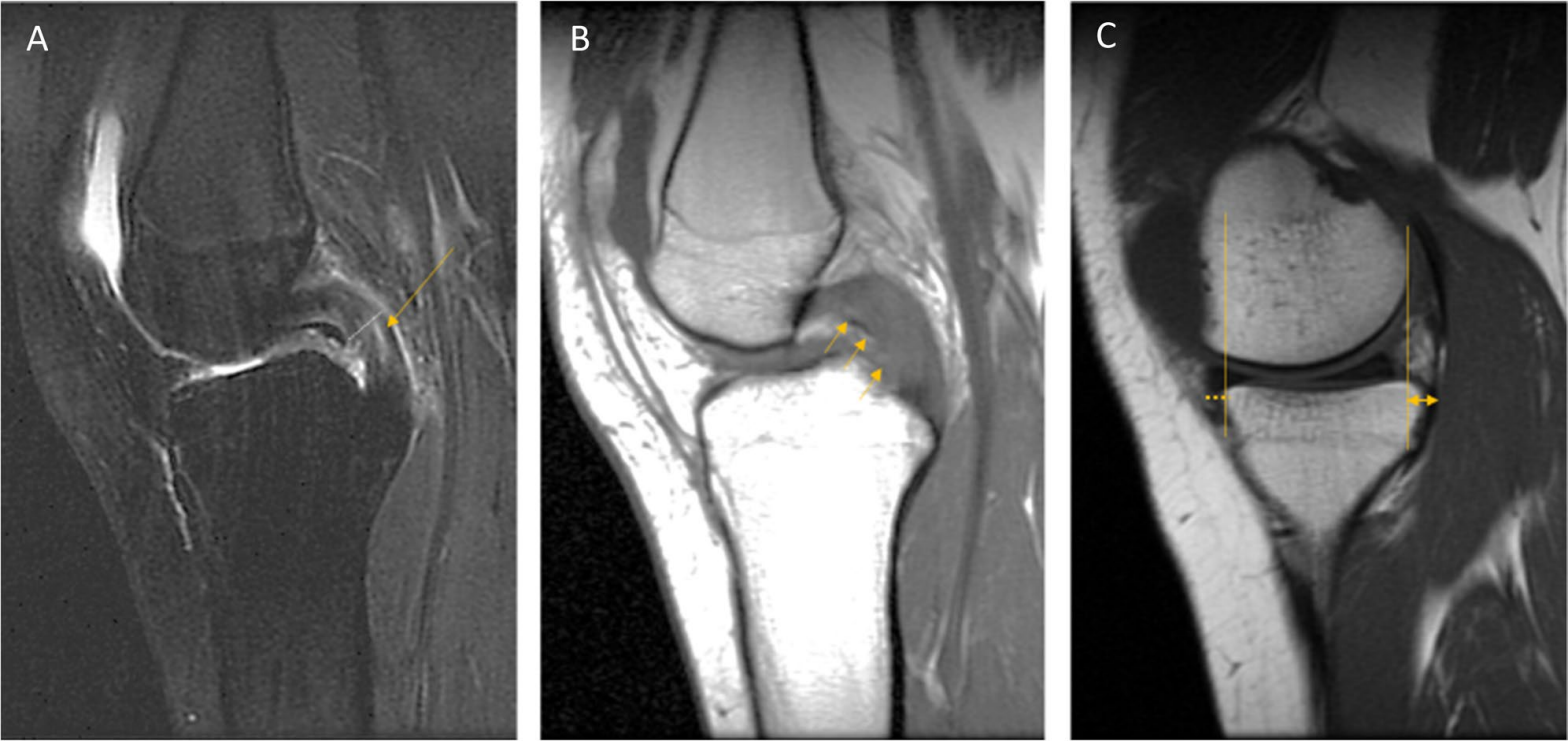
**A** PD sagittal image (with patient movement) shows high-signal intensity (arrow) in the PCL, which was also > 8 mm in thickness in the short axis. **B** T1 sagittal image, which shows intermediate-intensity signal in the PCL (arrow). **C** T1 sagittal image In the medial compartment, there is slight malalignment of the femur and tibia, where the tibia “sags” posteriorly > 10 mm, which makes the anterior medial meniscus protrude beyond the anterior rim of the tibia > 3 mm, each are by themselves an indirect sign of PCL deficiency

The four main stabilising ligaments of the knee are anterior (ACL) and posterior cruciate ligaments (PCL), and the medial (MCL) and lateral collateral ligaments (LCL). They provide stability to the knee during flexion–extension, valgus-varus movement, and internal and external rotation. In addition, the other capsular structures and muscle tendons, especially in the posteromedial and posterolateral corners, provide additional stability to the knee [22, 23]. Anterolateral capsular structures are considered important secondary rotational stabilisers in the knee [24]. All ligaments normally appear as dark signal structures on T1-, T2-, and PD-weighted images [19]. The cruciate ligaments are each two bundled which can be appreciated in all planes, and the ACL appears more striated than the PCL. This should not be misinterpreted as a partial thickness rupture of the ACL. Ligament pathology is seen as increased signal, thickening or thinning of the ligaments, or discontinuity of fibres (Fig. 2A–D). Bony avulsions at either the proximal or distal attachments of both the cruciate and collateral ligaments may also occur and should not be missed [24]. An associated avulsion is the Segond fracture, i.e. a small avulsion of the anterolateral tibial rim where the iliotibial band and anterolateral capsule attach. This is associated with an ACL rupture in adult patients. The arcuate complex is when the conjoined LCL and biceps femoris tendon attachment avulses the proximal fibular head, this findings is associated with an ACL rupture (Fig. 2D–E) [24]. The PCL differs from the ACL because it can appear to have intact fibres, but the knee may be PCL deficient. In addition to discontinuity of fibres, a complete PCL rupture is diagnosed when the short axis of the PCL is ≥ 8 mm. Both the ACL and PCL can show mucoid degeneration, which is seen as increased signal intensity in the centre of the ligament on T1-, T2-, and PD-weighted images. When the knee is extended, knee joint alignment is useful for assessing ACL or PCL deficiency in the sagittal plane. The tibia translates anteriorly in the lateral compartment in ACL deficiency; in PCL deficiency, the medial tibia moves posteriorly (> 10 mm) [11, 23]. A 3-mm anterior displacement of the medial meniscus in the mid-medial compartment on sagittal MRI can also be a sign of (chronic) PCL deficiency (Fig. 3A–C) [25]. *Level of evidence 3b/Low.*

We recommend reporting ligament pathology as full- or partial-thickness tears, bearing in mind the limitations of MRI in diagnosing partial tears [6, 26, 27]. Searching for indirect signs of ligament insufficiency or deficiency as well as evaluation of joint malalignment may increase confidence in diagnosis [23, 25]. *Level of evidence 5/Low* (Fig. 2A–D).

#### Cartilage and osteochondral lesions

**Fig. 4:**
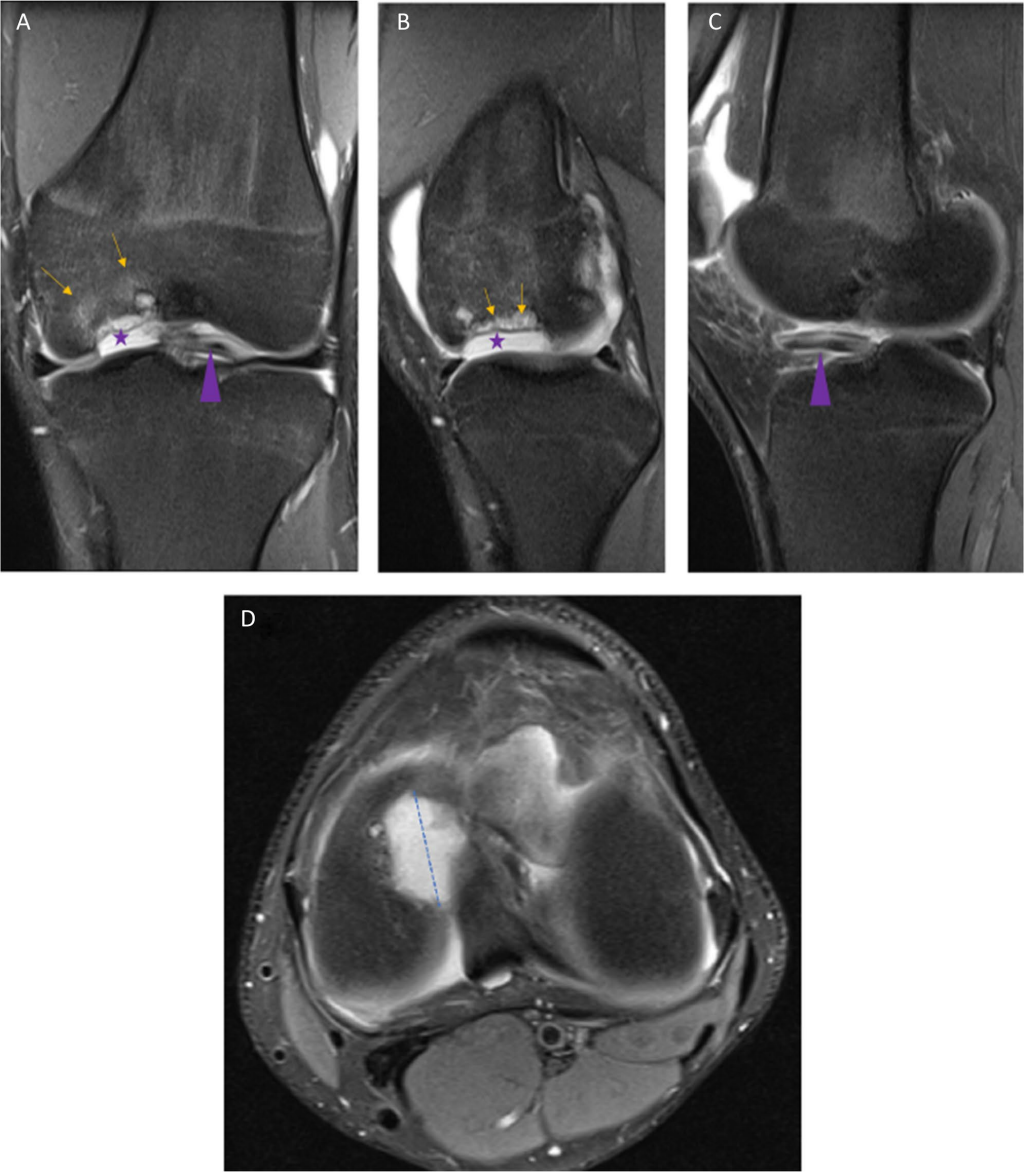
**A** PD coronal image after a previous fall shows an osteochondral defect (asterisk) with a dislodged osteochondral fragment (arrowhead). Slight oedema is also seen in the femur (arrows). **B** PD sagittal image shows the gap (star) left by the cartilage defect and the subchondral bone marrow oedema (arrows). **C** PD, the fragment seen in the sagittal plane (arrowhead). **D** PD, transverse plane, the largest size of defect best appreciated on this view, than on the other images, highlighting how one can underestimate the size of the cartilage defect, if one does not carefully evaluate all images

**Fig. 5:**
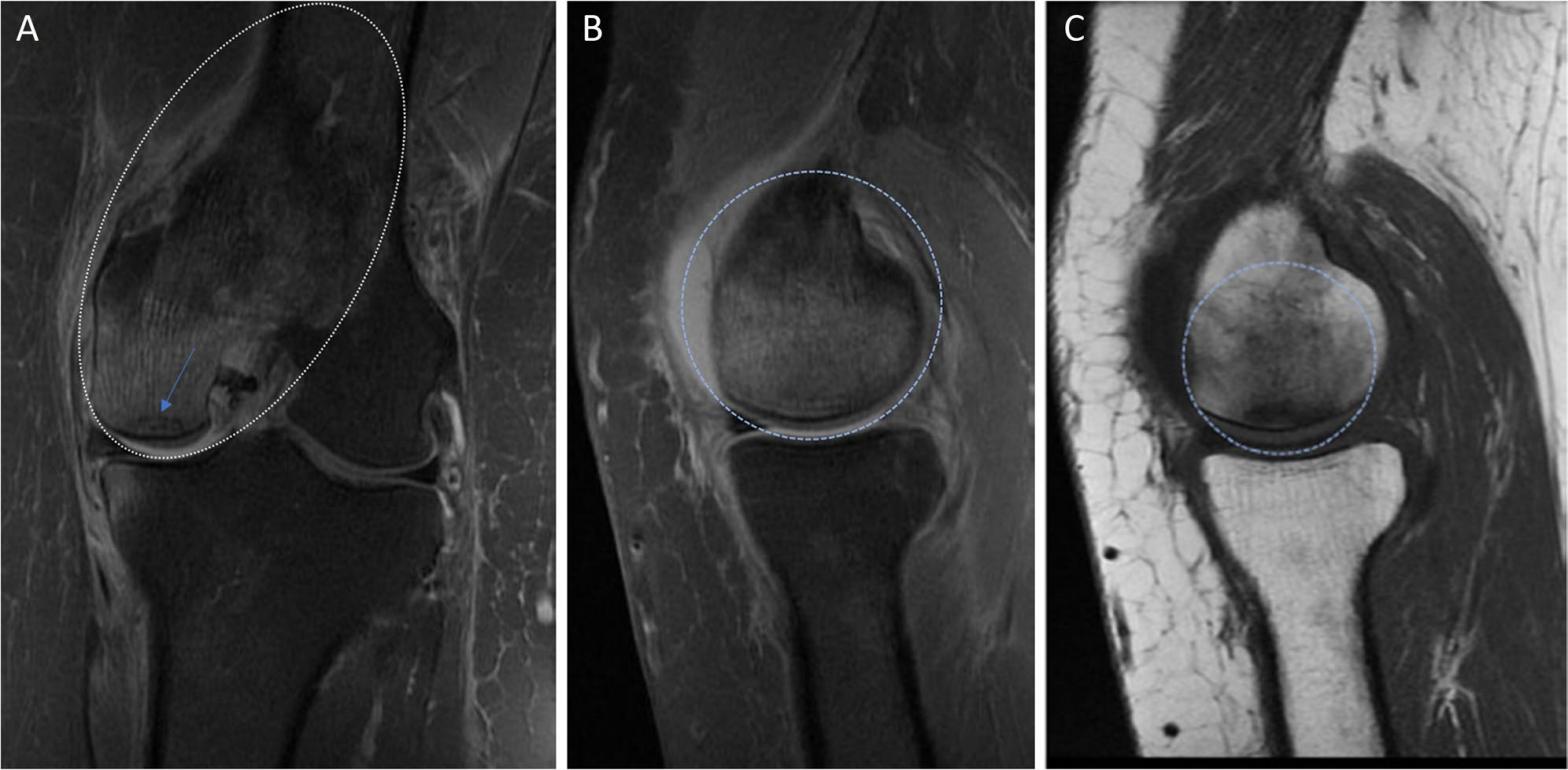
**A** PD (fat suppressed) coronal image shows extensive oedema in the medial condyle and distal femur metaphysis and diaphysis (dotted ellipse). A discrete subchondral low signal line is also seen (arrow). **B** PD sagittal image shows oedema in the femur (dotted circle), no certain subchondral line. **C** T1-weighted sagittal image shows the same oedema and a dark subchondral curved line, which represents a subchondral insufficiency fracture line

The femoral, tibial, and retropatellar surfaces are capped with articular cartilage [19]. Pathology is seen on fluid-sensitive sequences as high signal abnormalities. Cartilage pathology should be reported according to the depth of the lesion, graded as laceration/fibrillation when < 50%, and full thickness when > 50%, extending into the subchondral bone [7, 26]. One should be aware that MRI underestimates cartilage lesions [28]. A cartilage lesion can progress and affect the underlying bone and bone marrow, to an osteochondral lesion. The term osteochondral lesion encompasses several different entities [7]. In osteochondritis dissecans, there is disruption of endochondral ossification in the epiphysis, which results in an unstable fragment [7, 29]. If the fragment is displaced, it becomes an osteochondral defect (Fig. 4A–D). Any focal defect visible in the cartilage, as well as the underlying bone, may be called an osteochondral defect. Traumatic or insufficiency subchondral fractures (Fig. 5A–C), osteochondritis dissecans, avascular necrosis, and degenerative disease, all of which have different underlying pathophysiology, might progress into an osteochondral defect—the end state [29]. If one is uncertain which subgroup the lesion belongs to, one can safely use “osteochondral lesion(s)”; though it is an unspecific term, it is very accurate. Finally, it is not recommended to use the term “osteoarthritis” on MRI as this does not correlate with clinical findings, compared to osteoarthritis on radiographs which does correlate with clinical findings [7]. *Level of evidence 5/Low*.

MRI has a valuable role in differentiating stable from unstable osteochondral lesions in adults where the risk of a fragment loosening is related to visible high-signal intensity line between the bony fragment and rest of the bone [30].

#### Other structures

The infrapatellar fat pad (also called Hoffa’s fat pad) acts as a “protective pillow” and stabilises the anterior knee joint structures during function [31]. Fat pad oedema or fibrosis may cause symptoms [31]. Patellar maltracking is one of the causes for anterior knee pain and may be associated with ACL injury [31]. If this is suspected, further imaging findings indicating chronic patellar pathology on MRI include lateralisation of the patella and an abnormal sulcus depth or sulcus angle, as these findings render the knee joint susceptible to patellar subluxations [31].

**Fig. 6:**
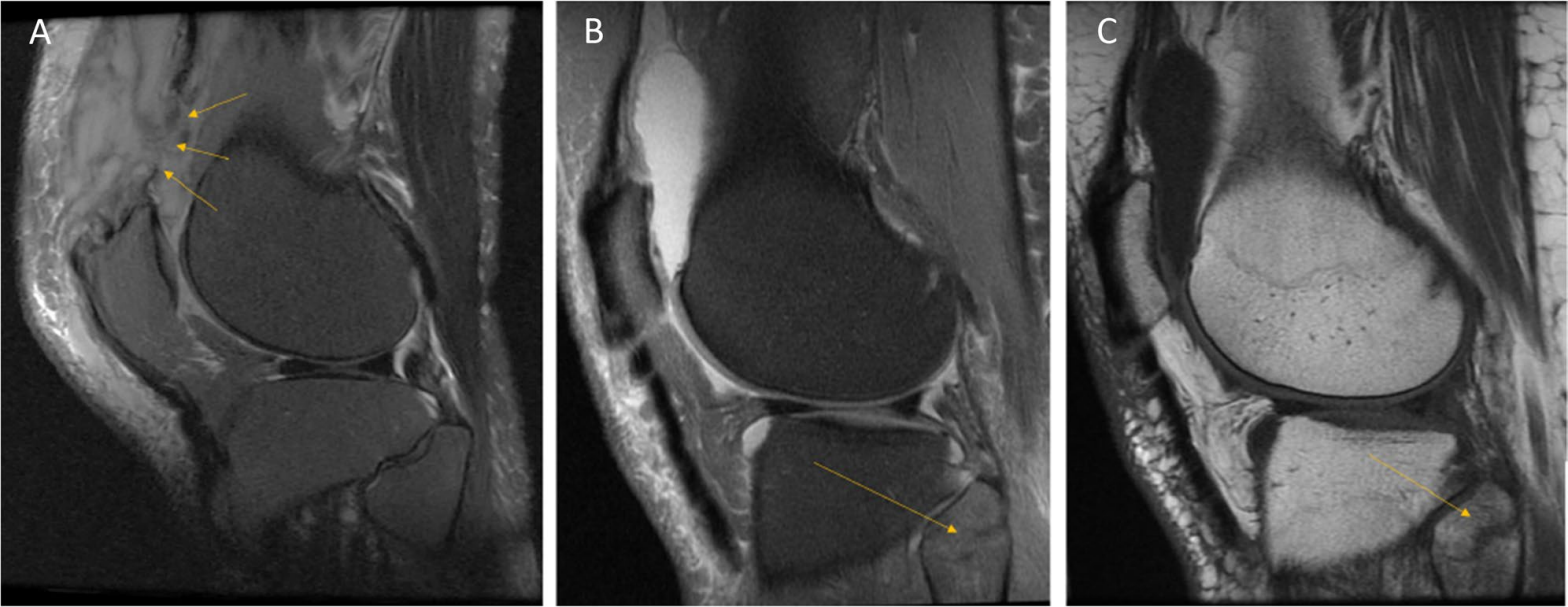
**A** PD (fat suppressed) sagittal image shows discontinuity of the quadriceps tendons after a direct fall on the knee (arrows). **B** PD (fat suppressed) sagittal image shows another patient who has had non-traumatic pain for more than 6 weeks, and subtle oedema, and a low signal line is seen in the fibular head (arrow). **C** On the T1-weighted sagittal image, the fracture line is much better appreciated (arrow). T1-weighted images are recommended in standard MRI of the knee, as not to miss unexpected but highly relevant findings

Rarely, soft tissue surrounding the knee may reveal neural ganglia or evidence of posttraumatic nerve injury and denervation of muscle compartments [32]. Finally, always look at all corners, as sometimes acute trauma of the tendons or fractures are not suspected clinically (Fig. 6A–C).

## Summary statement

MRI of the knee is commonly performed. The recommended MRI protocol includes both T1-weighted and fluid-sensitive images, in three orthogonal imaging planes. It is important to correlate the clinical findings with MRI findings; however, all structures from menisci, tendons and ligaments, bones, and soft tissues should be evaluated. It is important to use correct terminology, such as for partial- and full-thickness tear when reporting ligament pathology, for meniscal tears and lesions, and regarding osteochondral defects. It is also important to be aware of which pathology is usually underreported on MRI Table 1.

**Table 1:** List of the main recommendations and their corresponding levels of evidence [33, 34]

**Recommendation**	**Level of recommendation** [33]	**Level of recommendation** [34]	**References**
1. MRI protocol should include T1-weighted and fluid-sensitive images	4	Low	[8, 12]
2. Radiographs ought to be performed in addition to MRI in trauma	4	Low	[13]
3. Meniscal pathology should be reported according to the ESSKA recommendations	4	Low	[5]
4. MRI cannot definitely assess the stability of small meniscal tears	4	Moderate	[5]
5. MRI cannot exclude meniscal root tears	4	Moderate	[16, 19]
6. Ligament tears should be reported as partial- or full-thickness tears	5	Low	[6]
7. Cartilage defects should be reported according to depth of the defect and subchondral affection	5	Low	[7]
8. Knee alignment assessment can improve detection of PCL deficiency	3b	Low	[23]
9. The term “osteoarthritis” should not be used on MRI, as it includes clinical findings	5	Low	[7]

## Patient summary

MRI of the knee is a useful examination for assessing acute and chronic knee pain. Knee ligaments, menisci, bones, and soft tissue around the knee can be readily assessed. Radiographs serve as an adjunct to the MRI in the post-traumatic setting and are important so as to not miss subtle bony avulsions on MRI.

## Abbreviations

ACL: Anterior cruciate ligaments
LCL: Lateral collateral ligaments
MCL: Medial cruciate ligaments
PCL: Posterior cruciate ligaments
PD: Proton density

## Declarations

### Guarantor

The scientific guarantor of this publication is Anagha P Parkar.

### Statistics and biometry

No complex statistical methods were necessary for this paper.

### Methodology

• practice recommendations
